# Two Cases of Rare Ovarian Tumors

**Published:** 1890-05

**Authors:** Virgil O. Hardon

**Affiliations:** Professor of Obstetrics and Diseases of Women and Children, Atlanta Medica College, Atlanta, Ga.


### TWO CASES OF RARE OVARIAN TUMORS.

BY VIRGIL O. HARDON, M. D.,
Professor of Obstetrics and Diseases of Women and Children, Atlanta Medica College, Atlanta, Ga.

CASE I.—Sarcoma of Right Ovary. Ovariotomy. Recovery.—P. W., age 54, widow, multipara. Menopause occurred six years ago. Has always had good health until April, 1889, when she noticed a lump in the right side, which gradually increased in size. About two months later she noticed a general enlargement of the whole abdomen, which increased up to the time when she first came under my observation, in January, 1890. She had no pain at any time, her only inconvenience arising from the size of the accumulation in the abdominal cavity, which interfered with respiration and prevented her from lying down or walking about with any comfort. Her general health was not seriously impaired, although she had lost flesh.

When seen by me her abdomen was as large as that of a woman at the full term of pregnancy. External examination showed the abdominal cavity to be filled with ascitic fluid. In the right inguinal region could be felt a hard tumor, very movable, floating in the fluid and giving to the hand a very marked sensation of *ballottement*. Its attachment was evidently on the right side, as it could not be pushed beyond the median line. The tumor could also be felt from the vagina, but its attachment could not be made out. Diagnosis, solid tumor floating in ascitic fluid, the nature of the tumor not determined.

*Operation*, *Jan. 20*, *1890*.—Present, Drs. Kendrick and Harris and the class of students of the Atlanta Medical College. The abdomen was opened in the median line and a quantity of ascitic fluid evacuated, estimated at three gallons. The tumor, which was free from adhesions, was lifted out of the abdomen. It was attached to the right broad ligament by a slender pedicle consisting of the normal attachments of the ovary. The pedicle was transfixed and tied with silk. The abdomen was sponged out and about a pint of gelatinous material removed. No drainage. The abdominal incision was closed and dressed in the usual manner. The patient made a rapid recovery, although her temperature rose to 103.4 on the evening of the second day. She returned to her home completely cured on the 15th of February.

The tumor was oval in shape and had a smooth, white surface. On section it was found to be perfectly solid and closely resembled in appearance a fibroid tumor of the uterus. It weighed three and one-half pounds. At the point where the pedicle was divided on its lower side could be seen the remains of the ovary from whose surface the tumor appeared to grow. A microscopical examination was made by Dr. M. B. Hutchins, of Atlanta, who has kindly furnished me the following report: "I have examined sections both from frozen and from hardened portions of the specimen which you left with me. The disease is sarcoma and the tumor is made up of both small round and small spindle cells. Blood vessels are quite numerous in the round celled portions, while I could see none among the spindle cells, which were more closely packed together."

CASE II.—Superficial Papilloma of Both Ovaries. Ovariotomy. Recovery.—M. P., age 62, married, multipara. Menopause occurred at about 45 years of age. Health has always been good. In November, 1889, she first noticed a general enlargement of her abdomen. This enlargement increased with great rapidity, so that when she came under my care, early in March, she was larger than a woman at full term of pregnancy and was unable to stand up or lie down, but could only sit propped up in bed. She had no pain beyond the inconvenience arising from the bulk of the abdominal accumulation and, strange to say, her general health had not suffered apparently.

External examination showed the abdomen to be filled with ascitic fluid. No tumor could be felt through the abdominal walls. By vaginal examination an obscure mass could be made out in the pelvis, on each side of the uterus. Careful investigation failed to elicit any evidence of cardiac, renal or hepatic disease. There was no œdema of any part of the body. The urine was normal. No diagnosis was made. As the symptoms pointed to some diseased condition within the abdomen it was decided to make an exploratory incision.

Operation March 15, 1890.—Present, Drs. Harris, Murphey and Kennedy. The abdomen was opened in the median line and a large quantity of ascitic fluid evacuated. Upon exploring the cavity the surface of both ovaries was found to be completely covered with papillomatous growths, identical in appearance with cauliflower cancer of the cervix. The left ovary contained a cyst as large as an English walnut, entirely independent of the papilloma. There was no papillomatous growth within or upon the cyst. The right ovary was as large as a goose egg, while the left was as large as a man's fist. The disease was confined entirely to the ovaries, and there were no adhesions to neighboring organs. The peritoneum was normal as far as it was visible during the operation. Both ovaries were removed, the abdomen washed out and closed without drainage. The patient made a good recovery, her highest temperature being 100.2 on the evening of the first day.

No microscopical examination of these ovaries has yet been made, but their symptoms and gross appearances correspond exactly with Olshausen's description of superficial papilloma of the ovaries, of which he states that he has been able to find only six recorded cases. Such papillomatous growths are quite often found in the interior of a certain variety of ovarian cysts, but their occurrence upon the free surface of the ovary is one of the rarest of pathological conditions.

